# Endoplasmic Reticulum Stress in Metabolic Disorders

**DOI:** 10.3390/cells7060063

**Published:** 2018-06-19

**Authors:** Rose Ghemrawi, Shyue-Fang Battaglia-Hsu, Carole Arnold

**Affiliations:** 1 College of Pharmacy, Al Ain University of Science and Technology, 112612 Abu Dhabi, UAE; rose.ghemrawi@aau.ac.ae; 2 Nutrition Génétique et Exposition aux Risques Environnementaux, INSERM Unité 1256, Université de Lorraine, 54000 Vandoeuvre les Nancy, France; Shyue-fang.battaglia@univ-lorraine.fr

**Keywords:** endoplasmic reticulum stress, metabolic disorders, unfolded protein response, inflammation, lipotoxicity, glucotoxicity, therapy

## Abstract

Metabolic disorders have become among the most serious threats to human health, leading to severe chronic diseases such as obesity, type 2 diabetes, and non-alcoholic fatty liver disease, as well as cardiovascular diseases. Interestingly, despite the fact that each of these diseases has different physiological and clinical symptoms, they appear to share certain pathological traits such as intracellular stress and inflammation induced by metabolic disturbance stemmed from over nutrition frequently aggravated by a modern, sedentary life style. These modern ways of living inundate cells and organs with saturating levels of sugar and fat, leading to glycotoxicity and lipotoxicity that induce intracellular stress signaling ranging from oxidative to ER stress response to cope with the metabolic insults (Mukherjee, et al., 2015). In this review, we discuss the roles played by cellular stress and its responses in shaping metabolic disorders. We have summarized here current mechanistic insights explaining the pathogenesis of these disorders. These are followed by a discussion of the latest therapies targeting the stress response pathways.

## 1. ER Functions and Homeostasis

### 1.1. The Multiple Functions of ER in Protein, Lipid, and Calcium Homeostasis

The endoplasmic reticulum (ER) is the largest multifunctional organelle inside cells with distinct structure elements containing both smooth and rough ER. Some of the best-known ER functions include the production of lipids like cholesterol, glycerophospholipids, and ceramide, the synthesis of protein, and the regulation of calcium storage and dynamics [1]. As ER lumen is a highly oxidizing environment, it also provides the necessary setting for secretory protein folding and maturation [2]. However, ER folding processes are complex, and given the fact that as much as one third of the cell proteome may enter into ER for processing by molecular chaperones and enzymes, many of the newly synthesized proteins may never reach final native conformation due to error-prone folding processes. The presence of quality control pathways including unfolded protein response (UPR), ER-associated degradation (ERAD), and autophagy are thus necessary to maintain ER homeostasis [3,4].

### 1.2. ER Interconnections with Other Organelles 

ER is structurally and functionally connected with many other cellular organelles; thus, it is central to the integration of diverse signaling emanating from other compartments [5]. Structural connectivity is best studied at the level of plasma membrane (PM)-ER and mitochondria-ER junctions. The extensive ER network directly contacts the plasma membrane in numerous sites [6] involved in phosphatidylinositol metabolism, non-vesicular transfer of sterols, and calcium regulation [7,8,9]. In animal cells, the PM-ER junction is also part of the mechanism of store-operated calcium entry crucial for both short- and long-term intracellular calcium homeostasis needed for processes ranging from calcium-regulated secretion to gene expression [5]. The sites of ER in contact with mitochondria have been referred to as mitochondria-associated ER membrane (MAM). They contain microdomains enriched with lipids like cholesterol and ceramides that give rise to their particular structure [10]. The presence of MAM not only permits the exchange of lipids, metabolites, and ions between ER and mitochondria, it also provides the proper milieu to begin the synthesis of certain lipid synthesis like phosphotidylethanolamine (PE) and phosphotidylcholine (PC) [11]. MAM also contains particular protein elements to fulfill diverse cellular functions; for example, calcium-handling proteins like Inositol trisphosphate receptors (IP3Rs) are highly enriched in MAM, and their presence helps to attain interorganellar calcium equilibrium rapidly. Such rapid exchange of calcium into mitochondria is crucial for cell signaling, adaptation, and survival. In mitochondria, calcium regulates tricarboxylic acid (TCA) cycle via calcium-dependent dehydrogenases [12]; it impacts the production of adenosine triphosphate (ATP). Interorganellar calcium transfer between ER and mitochondria explains thus why ER-mitochondria coupling promotes mitochondria respiration during early phase of ER stress, but decreases mitochondria metabolism under prolonged ER stress due to calcium overload-induced apoptosis [13]. The pathological consequences of dysregulated MAM have been brought to light recently in various cell types. For example, in liver, obesity leads to a marked enhancement of MAM that alters interorganellar calcium dynamics and causes increased oxidative stress and metabolic derangement [14]. MAM disruption and miscommunication have also been implicated in muscle insulin resistance in mice and humans [15], and hepatic insulin resistance [16], respectively. Intriguingly, MAM is thought to be one of the sites of isolation membrane for the formation of selective autophagosomes for mitochondria [17]. Dynamic changes in either the composition or the number of MAMs are thus likely part of the cellular stress response designed either to combat cellular level insults or to terminate unsolvable cellular hardships by self-destruction (e.g., autophagy, apoptosis). Incidentally, ER makes additional contacts with Golgi apparatus likely via proteins similar to yeast Nvj2p, which tethers ER to Golgi apparatus. This appears to regulate ceramide transfer to avoid toxic accumulation of these lipids [18]. 

## 2. ER Stress and the Unfolded Protein Response (UPR)

### 2.1. The UPR

**Figure 1:**
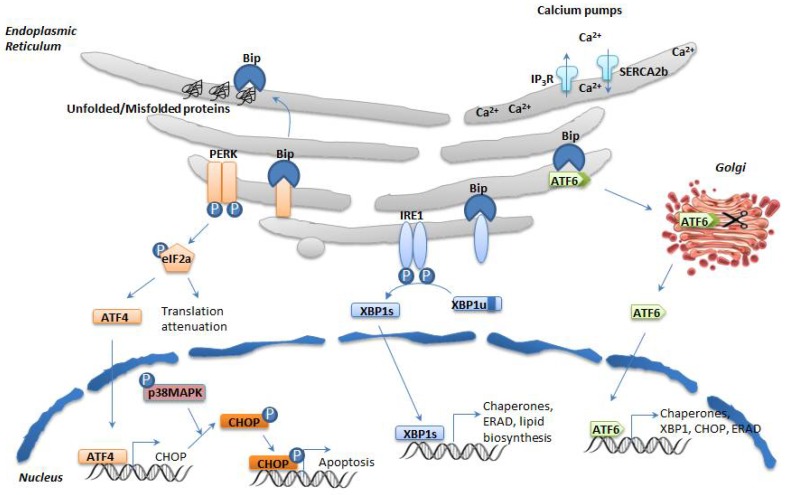
ER stress and the UPR. IP3R receptor and calcium pump SERCA are responsible for the efflux and influx of calcium in ER, respectively. Intracellular calcium dysregulation induces ER stress. ER stress induces the UPR. Unfolded or misfolded proteins are recognized by Bip, which then releases itself from PERK, IRE1, and ATF6, leading to their activation. The activation of PERK involves its homodimer formation and autophorsphorylation. It subsequently phosphorylates eIF2α, which in turn attenuates general translation but facilitates the translation of ATF4, thus increasing its protein level. ATF4 is a transcription factor that activates the transcription of CHOP. Phosphorylated p38 MAPK phosphorylates CHOP, which then triggers the transcription of apoptotic genes. Similar to PERK, IRE1α activates after the formation and autophosphorylation of IRE1α homodimers. This activates IRE1α RNase activity. IRE1α then splices *Xbp1*, removing 26 nucleotides from its transcript. Then, the XBP1s spliced form of XBP1 acts as a transcription factor for chaperones, as well as genes involved in ERAD and lipid biosynthesis. ATF6 activation involves its release from Bip, and its translocation into the Golgi, where it undergoes cleavage by S1P/S2P proteases. This allows its nuclear translocation and subsequent transcriptional activation of chaperones, XBP1, CHOP, and ERAD genes. Abbreviations: IP3R (inositol 1,4,5-trisphosphate(IP3R) receptor).

In mammalian cells, the UPR is controlled by three transmembrane mediators: RNA-dependent protein kinase (PKR)-like ER kinase (PERK), Inositol- requiring enzyme 1α (IRE1α), and activating transcription factor 6 (ATF6). Under normal conditions, these proteins are associated with Bip or GRP78 (78 KDa glucose-regulated protein) and thus remain inactive. Under ER stress, Bip is released from these sensor proteins, and UPR cascade is activated after further dimerization and autophosphorylation of PERK and IRE1α, and regulated intramembrane proteolysis of ATF6 (Figure 1). PERK activation leads to eukaryotic initiation factor 2α (eIF2α) phosphorylation, reducing thus global protein synthesis consequent to the reduced and slower formation of the eIF2-containing initiation complex; this change triggers also the translation of activating transcription factor 4 (ATF4) thanks to a longer resident time on its first uORF of the initiation complex of translation. ATF4, in turn, up regulates pro-apoptotic factor C/EBPα-homologous protein (GADD153 or CHOP). CHOP subsequently regulates the balance between proapoptotic and antiapoptotic B-cell lymphoma (BCL)-2 family proteins; for example, it mediates the direct transcriptional induction and translocation to the ER membrane of Bim, a proapoptotic BH3-only protein of the BCL-2 protein family in conditions of prolonged ER stress [19].

IRE1α is the second player of the UPR. The activation of IRE1α after its dimerization and autophosphorylation triggers its endoribonuclease activity, which enables an unconventional splicing of the X-box binding protein 1 (*Xbp1*) mRNA through cleaving off a 26-nucleotide sequence. This results in a frame shift of the XBP1u, producing eventually an active transcription factor (XBP1s) [20]. IRE1α constitutes one important player of the UPR: upon short term stress, via XBP1s, IRE1α upregulates the expression of chaperones and ERAD, and induces cell survival by inhibiting CHOP; under prolonged stress, IRE1α promotes cell death by activating JUN N-terminal kinase (JNK) [21]. In addition, IRE1α interacts with the adaptor protein tumor necrosis factor TNF receptor associated factor 2 (TRAF2), activating thus the apoptosis signal-regulating kinase (ASK1) [22] and caspase 12 [23]. The third transmembrane mediator of UPR is ATF6. ER stress induces the release of Bip from ATF6, permitting its translocation from ER to Golgi, where S1P- and S2P-proteolytic cleavage produce a transcriptionally active cytosolic fragment that regulates the expression of XBP1 and induces the transcription of proteins related to protein folding and ERAD. ATF6 has 2 isoforms: ATF6α and ATF6β. Both isoforms have conserved DNA-binding domains but divergent transcriptional activation domains; ATF6α is a strong transcriptional activator, whereas beta is a weak activator [24]. 

Besides activating these three branches of the UPR, ER stress also disturbs the communication between ER and other organelles. A recent study established that upon pathologic ER insults, PERK integrates transcriptional and translational signaling to coordinate mitochondrial protection. The UPR is involved in preventing pathologic mitochondrial fragmentation and promoting mitochondrial metabolism in response to ER stress [25]. Upon ER stress, the ER morphological structure is altered, including luminal swelling and dissociated ribosomes [26], and it is assumed that the contacts with other organelles are compromised. Overall, UPR mediators are involved in maintaining ER homeostasis. When adaptive responses fail to resolve ER stress, UPR initiates regulated cell death.

### 2.2. ER Senses Cell Stress and Integrates Metabolic Stress Signals 

**Figure 2:**
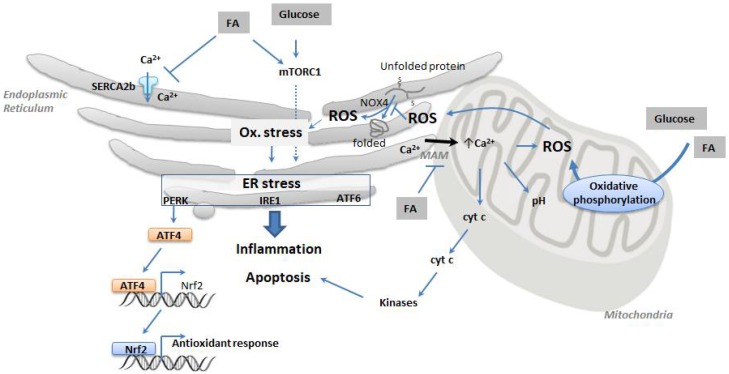
ER and the mitochondria function together as sensors of nutrient excess and integrators of the response to metabolic stress. Reactive oxygen species (ROS) are free radicals that are generated as a by-product of cellular metabolism from FA and glucose in the mitochondria. FA enhance MAM; thus, excessive cytoplasmic calcium enters in mitochondria and ER, triggering changes in mitochondrial pH and ROS production. This alters mitochondrial membrane potential and opens pores, thus releasing cytochrome c. Several calcium-dependent proteins and kinases are then activated, triggering apoptosis. ROS perturb the redox status of ER lumen and thus inhibit protein folding. The ER-associated protein NADPH oxidase 4 (NOX4) is implicated in ROS generation during disulfide bond formation for proper protein folding. Excess ROS cause oxidative stress, which induces the 3 branches of the UPR. The result of the UPR activation is inflammation and, upon sustained and/or intense stress, apoptosis. FA also disrupt SERCA activity and perturb ER calcium homeostasis. The mTORC1 complex is an important sensor of bioenergetic status and nutrient excess, and induces ER stress by unclear mechanisms. PERK-mediated activation of ATF4 induces NRF2, a transcription factor responsible for antioxidant cell response. Abbreviations: cyt c, cytochrome c (cyt c); Ox. stress, oxidative stress (Ox. Stress); MAM, mitochondria-associated membranes (MAM); mTORC1, mammalian target of rapamycin complex 1 (mTORC1); NOX4, NADPH oxidase 4 (NOX4); ROS, Reactive oxygen species (ROS); SERCA, sarco-endoplasmic reticulum Ca(2+)-ATPase (SERCA). Sharp arrows, activators; bar-ended arrows, inhibitors.

Metabolic stress is a condition characterized by insufficient or excessive nutrient supply compared to cellular bioenergetic needs. Excessive supply is characteristic of over nutrition that represents a public health concern in developed and developing countries. Chronic metabolic stress leads to a variety of metabolic diseases, including obesity, insulin resistance, diabetes, fatty liver disease, and cardiovascular diseases. The two main types of nutrients that exert cytotoxic effects via their action on ER are fatty acids (FA) and glucose (Figure 2).

Peripheral tissues satisfy their energy needs in part by oxidizing the fatty acids liberated from triglycerides originating from dietary lipids. Excessive fatty acids are stored in adipocytes to provide energy during fasting periods. However, when the supply of FA overwhelms the storage capacity of adipose tissues, circulating FA rises. As a result, lipids accumulate in cells/tissues lacking adequate molecular machinery for their conversion and storage, thus damaging the cells. This is particularly true for saturated long-chain FAs like palmitate (C16:0, the most commonly consumed FA) and stearate (C18:0), although unsaturated fatty acids like oleate (C18:1) appear mainly protective and can in some instances partially counteract the effects of saturated FA. Recent evidence suggests that the lipotoxic effects of saturated FAs like palmitate may be linked to its excessive incorporation into membranous structures like ER, hence reducing the presence of sphingomyelin and cholesterol that form part of ER lipid rafts needed for cargo loading to secretory vesicles for export to Golgi [27]. Lipotoxic effects on ER may also be triggered by high levels of circulating free cholesterol, as its uptake into membrane alters the composition and hence the stability of this low-cholesterol containing organelle [28] (for lipotoxicity triggered by other categories of lipids, see recent reviews elsewhere including [29]). Consistent with its high sensitivity to cholesterol level, ER also regulates cholesterol metabolism via ER-resident transcription factor nuclear respiratory factor 1/nuclear factor, erythroid 2 like 1 (Nrf1/Nfe2L1), a guardian of cholesterol homeostasis [30]. Lipotoxicity at the level of ER can thus have functional consequences on ER resident proteins like ER stress sensors [31], molecular chaperones, and enzymes like sarco-ER Ca(2+)-ATPase (SERCA) [32]. 

Besides lipotoxicity, glucotoxicity due to chronic exposure to sugar-enriched dietary intake constitutes another major insult to cells. Excessive sugar affects organs both structurally and functionally, damaging vasculature, as well as kidneys, retina, and peripheral nervous system. Among different forms of sugars, it is worth noting that glucose is not the sole carbohydrate to be cytotoxic: high-level fructose or sucrose also induces insulin resistance in rodent [33]. Within cells, glucotoxicity enhances N-linked protein glycosylation occuring co-translationally in the ER (reviewed in [34]). Interestingly, glucotoxicity may have less obvious ER effects than does lipotoxicity [35], and predominantly induces oxidative stress [36]. Oxidative stress often induces ER stress, as there is a tight crosstalk between the two; for example, the ROS produced by disulfide bond formation causes both oxidative and ER stress. However, some authors reported that glucotoxicity-induced ER stress was independent of oxidative stress, and that antioxidant therapies alleviated oxidative stress without suppressing ER stress in a diabetic context [37].

These two arms of nutrient toxicity might also act in synergy, a concept termed ‘glucolipotoxicity’, in which glucose exacerbates the toxic effects of lipids via the production of potentially harmful lipid intermediates. The mechanisms are still unclear; however, it is thought that glucolipotoxicity is an aggravated form of lipotoxicity, in which glucose amplifies the deleterious effects of lipids [38]. ER dysregulation might be an important mechanism of glucolipotoxicity, and this has been reviewed in detail elsewhere [39]. Mammalian target of rapamycin complex 1 (mTORC1), an important sensor of bioenergetic status with a role in cellular stress regulation, has been proposed as an important ER stress transducer of glucolipotoxicity in beta cells. This mTOR complex regulates the activation of IRE1 and JNK by ER stressors [40]. As ER is the site of metabolically sensitive modifications of proteins, ER stress response thus gives ER its capacity to nutrient sensing. Altogether, glucose and lipid excess is cytotoxic, and their combined detrimental effects are mediated through key nutrient sensors, as well as ER stress.

ER and the mitochondria have partially overlapping functions, and there is strong crosstalk between the two organelles to sense states of cellular stress like oxidative stress and inflammation. In fact, ER and mitochondria are gaining recognition as key integrators of signals arising from different stress pathways to produce adequate response and eventually regulate cell death, the final outcome when stress cannot be resolved. Interestingly, these two organelles together produce the large majority of the cellular reactive oxygen species (ROS) [41]. In mitochondria, ROS production is long thought to be the byproduct of oxidative metabolism linked to ATP production, whereas in ER, ROS is generated during protein folding as a result of disulfide bond formation. The ER-associated protein NADPH oxidase 4 (NOX4) is implicated in ER ROS generation [42]. Excess ROS can overwhelm the anti-oxidative capacity of cells, leading to oxidative stress. This is particularly true for cells like pancreatic beta cells bearing only limited level of intrinsic antioxidant defense that are thus more prone to oxidative stress. In mitochondria, the main source of ROS is generated by oxidative phosphorylation. ROS produced by mitochondria represents a potent inducer of ER stress through disturbing the redox status of ER lumen [43]. The main functional link between mitochondria and ER involves calcium flow. Cytoplasmic calcium rise is normally taken up into mitochondria and ER; in mitochondria, this alters mitochondrial membrane potential and changes mitochondrial pH, affecting downstream production of ATP and ROS; under conditions of prolonged stress, it also opens permeability transition pores, causing cytochrome *c* leakage into cytoplasm. On one hand, in ER, the high calcium content can further flow into mitochondria through IP3R enriched MAM to stimulate mitochondria respiration and ROS generation. On the other hand, in mitochondria, ROS, after arriving in ER, deregulates ER resident calcium channels and causes massive ER calcium release into cytoplasm, fueling thus further mitochondria ROS production [44]. ER stress response and oxidative stress signaling coordinate further via PERK-mediated activation of ATF4 and nuclear factor erythroid 2–related factor 2 (NRF2)—the latter being a transcription factor responsible for antioxidant cell response [45]. ER stress response thus interacts with mitochondria stress response via calcium and ROS/anti-oxidative signaling. ER stress and oxidative stress can thus become locked in a vicious cycle, each forcing the other higher and higher, aggravating the final pathological outcome.

Incidentally, the interplay between ER and oxidative stress pathway often leads to the activation (via transcription factors such as Nf-kB, AP1, and STAT3 signaling pathway) of inflammation, a key manifestation of metabolic disorders (reviewed in [46,47]). Interestingly, inflammation can activate UPR through PERK, IRE1, and ATF6 signaling, and UPR, in turn, can regulate key proinflammatory pathways involving the nuclear factor κB (NFκB) and JNK/Activator protein 1 (AP1) [48]. For instance, the NFκB pathway can be activated by all three branches of UPR, while the JNK/AP1 is mainly triggered by IRE1. This crosstalk of ER stress and inflammation thus feeds on itself in a vicious cycle to worsen the metabolic syndromes and leads to cell death.

In summary, ER participates to the integration process of all the key metabolic signals (calcium signaling, nutrient toxicity, and oxidative stress), leading to inflammation and eventually to cell death.

## 3. ER Stress Induces IR and Diabetes 

**Figure 3:**
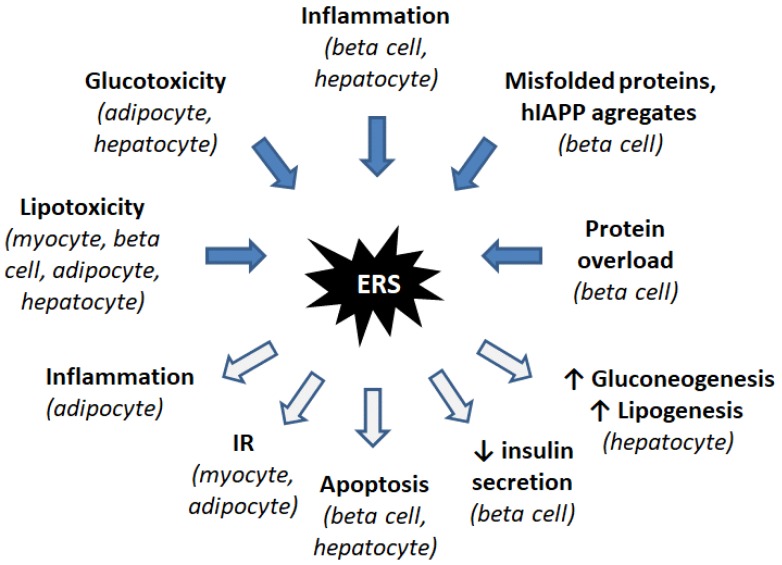
Induction and consequences of ER stress in insulin resistance and diabetes. In diabetes, lipotoxicity and glucotoxicity induce ER stress in several cell types. Inflammation is another inducer of ER stress. In beta cells, intense insulin production leads to misfolded insulin and hIAPP aggregates, which induce ER stress. ER stress triggers various responses, including inflammation, IR, apoptosis, decrease of insulin secretion, and increase of gluconeogenesis and lipogenesis, depending on the considered cell type.

Chronic metabolic stress induces both ER and oxidative stress and is invariably associated with inflammation, an element of cellular stress response considered as a major cause of obesity, insulin resistance (IR), and type 2 diabetes. These pathologies are characterized by a general multi-organ dysfunction, including liver, muscle, adipose tissue, brain, and pancreas, and ER stress is associated with the dysfunction of these tissues. Some of the ER-linked mechanisms are common to all these tissues, while others are cell type-specific (Figure 3). 

### 3.1. Importance of the ER in IR and Diabetes

**Figure 4:**
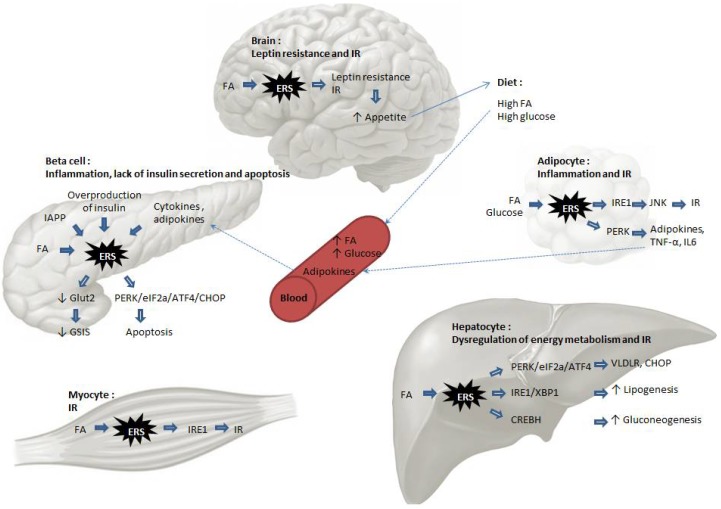
ER stress and the UPR in insulin resistance and diabetes. Insulin resistance and diabetes involve dysregulations in multiple organs, and ER stress participates to all these dysregulations. Excessive dietary FA and glucose induce ER stress in the brain, beta cells of Langerhans islets, myocytes, hepatocytes, and adipocytes. In the brain, this triggers insulin resistance and leptin resistance, with the latter enhancing appetite and thus excessive nutrient intake. In hepatocytes, FA-induced ER stress induces the three branches of the UPR. The PERK arm induces VLDLR and CHOP; the IRE1 arm increases lipogenesis and the CREBH arm enhances neoglucogenesis. In myocytes, this induces the IRE1 arm of the UPR, which deregulates the insulin receptor signaling and thus leads to insulin resistance. In adipocytes, FA and glucose trigger the IRE1 and PERK pathways, which induce, respectively, insulin resistance via JNK induction, and adipokines, TNFα, and IL-6 secretion. These adipokines and cytokines are released into the bloodstream and trigger ER stress in beta cells. FA, IAPP, and insulin overproduction are other triggers of ER stress in this cell type. Consequences of ER stress in beta cell are apoptosis through the PERK/eIF2α/ATF4/CHOP pathway and decreased GSIS via GLUT2 repression. Abbreviations: ERS, ER stress; GSIS, Glucose-Stimulated Insulin Secretion; IR, Insulin Resistance.

The importance of ER stress response in diabetes is highlighted by the finding that mouse mutants for PERK exhibit beta-cell loss and diabetes [49]. Moreover, in humans the mutation of PERK leads to a rare genetic disease named Wolcott-Rallison syndrome, characterized by insulin-dependent diabetes [50]. In contrast, CHOP deletion alters neither glucose tolerance nor insulin sensitivity [51]. ATF6α-null mice display beta cell function impairment upon high fat diet but no such diet-induced insulin resistance; thus, ATF6α has ambivalent role on diabetes development [52]. These findings implicate ER dysfunction to insulin signaling and diabetes. We will now describe the pathways involved and the molecular mechanisms (Figure 4).

### 3.2. Inflammatory Response 

Inflammatory response is a protective immune response to either internal or external insults like cell/tissue damages or infections aimed to remove the cause and to repair the damages. Its beginning is marked by the release of inflammatory substances like cytokines, free radicals, and hormones. In the context of ER stress, it is known that while a chronic presence of excessive metabolic factors such as lipids (the so called lipotoxicity) and sugars (the so called glucotoxicity) triggers inflammation, the same circumstances trigger also ER stress response, which can both activate and aggravate the inflammatory response. For example, in the case of obesity, it has been associated with chronic low-grade inflammation, but the mechanism linking inflammation to obesity remains unclear. ER stress is increasingly recognized as a mediator between glucolipotoxicity and inflammation in adipocytes. In adipocytes, saturating level of fatty acids induces ER stress via ROS generation [53]. Chemical chaperones 4-phenylbutyrate (4-PBA) and Tauroursodeoxycholic acid (TUDCA) attenuate the inflammatory response and the metabolic perturbation in adipocytes of obese mice [54]. Thus, ER stress plays a role in inducing the inflammatory response in adipocytes, probably via NFκB pathway that leads to the secretion of Tumor necrosis factor alpha (TNF-α), interleukin-6 (IL-6), and IL-1β. More specifically, obesity-linked ER stress is present in brown adipose tissue, which appears to enhance the expression of TRIP-Br2, a transcription regulator that inhibits lipolysis and thermogenesis [55]. In visceral fat, ER stress induces TRIP-Br2 expression via the transcription factor GATA3 [56]. White adipose tissue has been the most extensively studied, but similar defects involving inflammation and oxidative stress are also seen in brown adipose tissue in a context of high fat diet-induced obesity [57]. Very recently, a novel ER membrane protein, the transcription factor NFE2L1, was implicated in adaptation to obesity, and its deletion in brown-adipocyte-resulted in ER stress and tissue inflammation [58]. Inflammation of adipose tissue is a crucial step towards insulin resistance. Very recently, it has been shown that retinoic acid receptor-related orphan receptor α (RORα), which is involved in inflammation in some tissues, induces ER stress in adipocytes, and that this can be attenuated by treatment with chemical chaperones [59]. Obesity also reduces the differentiation of preadipocytes into adipocytes, rendering these immature cells more prone to cytokine secretion. Recently, it has been shown that CHOP down regulates Th2 response cytokines and alters the microenvironment of adipocytes, thus affecting the function of macrophage within adipose tissues [60]. 

Some interleukins induce oxidative and ER stress via Janus kinase–signal transducer and activator of transcription-NFκB (JAK-STATs-NFκB) pathway. IL-23, IL-24, IL-33 are elevated in diabetic beta cells and are the most potent interleukins in ER stress activation [61]. In obese mice, antibodies neutralizing IL-23 or IL-24 reduce this stress, opening a way for therapeutic manipulation. However, other cytokines like IL-22 play a protective role [62]. In beta cells, adipocyte-secreted hormone leptin induces IL-1β secretion and represses the secretion of its natural antagonist IL-1Rαa [63]. IL-1 signaling in diabetic islets activates the innate immune system [64]. IL-1β has been shown to exacerbate inflammatory response in beta cells via the IRE1α/XBP1 pathway [65]. Palmitate leads to NFκB activation and ER stress in beta cells and, notably, to IL-1β production [66]. An expression analysis of cytokine-induced genes revealed that TNF-α and Interferon gamma (IFN-γ) induce ER stress in pancreatic islets, and that nitric oxide (NO) production is only involved in rats, not in mice or humans [67]. In beta cells, cytokine-induced ER stress induces autophagy, as an attempt to restore energy homeostasis. Interleukins bind surface receptors on beta cell and activate the well-known intracellular JAK-STAT pathway that triggers a transcriptional response, leading to ER stress and cell death. This leads to a local inflammation context. The leucocyte recruitment and activation are evident in type 1 diabetes (T1D) but not in type 2 diabetes mellitus (T2DM), in which the prominent mechanism of inflammation is the activation of resident macrophages and of adipocytes themselves.

Altogether, while metabolic derangement can trigger ER stress which, in turn, induces inflammation, it can also activate directly the proinflammatory cytokine expression, and, as a result, causes ER stress response (reviewed in [68]). In either case, the key players are JNK/AP1 and NFκB; the crosstalk point of the ER and inflammatory responses as JNK/AP1 and NFkB pathway can be activated by either cytokine signaling or ER stress. Incidentally, in ER stress response cascade, JNK/AP1 pathway is activated by IRE1, while NFκB pathway is activated by PERK/eIF2α, IRE1, and ATF6. IRE1/XBP1 also activates proinflammatory response. Unexpectedly, in liver, inflammation has beneficial effects on glucose homeostasis via XBP1s phosphorylation by IκB kinase β (IKKβ) [69].

### 3.3. Lipotoxicity

ER is involved in lipid metabolism, and lipotoxicity is one of the most important triggers of ER stress in peripheral tissues. Although lipid toxicity on various cell types has been described for a long time, the mechanisms are still unclear, and ER stress might represent a relatively new explanation for these deleterious effects. Damage of beta cells by prolonged exposure to circulating FA is considered as a main cause of diabetes. High fat diet by itself is not sufficient to induce diabetes, but it contributes to the worsening of the diabetic condition in genetically predisposed individuals. Upon excessive nutrient supply, the FA released by the adipose tissue triggers ER stress in peripheral tissues at least via two arms of the UPR, namely, the PERK-eIF2α-ATF4 and IRE1-XBP1 pathways. Dietary supplementation with palmitate for 2 weeks upregulated ER stress markers in mouse Langerhans islets and suppressed glucose-stimulated insulin secretion (GSIS). CHOP, Bip, and XBP1 transcripts were all upregulated [70]. MIN6 β-cells are exposed to palmitate undergo apoptosis, which is potentiated by XBP1 inhibition [71]. ER calcium depletion has also been identified as a mechanism of FA toxicity via the activation of IRE1, PERK, and ATF6 pathways [35]. ER stress and islet dysfunction is alleviated by the overexpression of NAD+-dependent protein deacetylase Sirtuin-3 (SIRT3) [72]. RNA sequencing in human pancreatic islets treated with palmitate identified ER stress as a major consequence of lipotoxicity, and functional studies pointed out novel mediators of adaptive ER stress signaling [73]. Interestingly, inhibition of calcium influx alleviates ER stress and attenuates beta cell dysfunction and death [74]. In skeletal muscle, palmitate disturbs lipid distribution, thus causing ER expansion and stress, finally leading to impaired insulin responsiveness, which is alleviated by oleate [75]. Oleate activation of the AMP-activated protein kinase (AMPK) has been proposed as a mechanism for this alleviation [76]. In adipocytes, ER stress triggers lipolysis by activating the kinases cyclic AMP/Protein kinase A (cAMP/PKA) and extracellular signal–regulated kinases (ERK1/2), and by enhancing the activity of cellular lipases, thus regulating energy homeostasis. This may contribute to dyslipidemia and lipotoxicity [77]. In brain, palmitate-induced CHOP activation decreases insulin-like growth factor 1 (IGF1) and leptin expression in vitro and in vivo; these two peptide hormones have essential implications in a variety of metabolic diseases [78]. Beside long-chain fatty acids, other lipids like cholesterol, diacylglycerols, phospholipids, and ceramides exert deleterious effects. For example, in beta cells, cholesterol exerts its toxic effects by activating Bip, eIF2α, ATF4, and CHOP, and induces autophagy to protect the cells from ER stress-induced damages [79].

### 3.4. Glucotoxicity

Excessive glucose levels are known to impair cellular metabolism and activate cellular stress signals including oxidative stress, ER stress, and mitochondrial dysfunction. One of the aspects of glucotoxicity is its ability to activate IL-1β secretion by beta cells, thus linking glucotoxicity to inflammation and impairing islet cell function, and survival. Glucotoxicity also has indirect effects on ER, via protein succination by fumarate produced by mitochondria under excess glucose levels. The ER-resident oxidoreductase protein disulfide isomerase (PDI) is succinated in adipocytes cultured with high glucose levels, with negative effects on its activity [80]. In cultured rat and mouse islets, high glucose induced the expression of heat shock 70 kDa protein 5 (HSPA5) and several other genes involved in ER stress. Beta cells are particularly sensitive to ER stress, as they express high levels of proinsulin, which is maturated in the ER. Therefore, ER normal function is vital for this cell type, as assessed by the fact that prolonged UPR leads to cell demise and diabetes in human and rodents [81,82]. Several studies reported in vitro or in vivo activation of UPR genes by excessive glucose levels (reviewed in [83]). Moreover, genetic manipulation of UPR components interferes with glucotoxic response. Silencing of insulin gene expression is impaired by *Atf6* disruption in INS1 cells [84]. Rat beta cells with *Xbp1* overexpression display lowered GSIS and increased apoptosis [85]. In contrast, other studies reported that high glucose intake in humans induces oxidative stress and carbonylation of glucose transporter-4 (GLUT-4) in adipocytes but not ER stress or inflammation [86]. Thus, this point deserves further investigation. Interestingly, fructose seems to be as detrimental as lipids on ER stress in liver and pancreas, leading to insulin resistance and diabetes as well [87]. 

### 3.5. Insulin Receptor Modulation

ER stress-induced insulin resistance stems in part from the disruption of insulin signaling in insulin-responsive cells. The mechanisms include hyperactivation of JNK pathway and subsequent serine phosphorylation of insulin receptor substrate-1 (IRS-1). Phosphorylation of IRS1 at Ser307 strongly correlates with insulin resistance in diabetic mouse and humans. Insulin resistance in turn leads to glucose intolerance. The importance of ER stress in this process is highlighted by the fact that XBP1-null mice develop insulin resistance [88]. Genetic beta cell disruption of IRS1 but not IRS2 decreased XBP1s level and cell death [89]. IRS1 might protect beta cells against ER stress-induced apoptosis by modulating XBP-1 stability, protein synthesis, and calcium storage in the ER. In rat insulinoma cells, palmitate induces ER stress, phosphorylation of eIF2α, JNK, and IRS1, which is alleviated by the well-known insulin sensitizer metformin [90]. MTORC1-induced insulin resistance in mice has been shown to occur via ER stress activation [91]. In HepG2 cells and in cultured adipocytes, the novel ER stress-reducing chemical chaperones hydroxynaphthoic acids (HNAs) reduced palmitate-induced phosphorylation of JNK, IKKβ, and IRS1 (S307) and restored insulin signaling cascade which involves insulin receptor β, IRS1, and Akt. Moreover, in vivo HNA treatment restored glucose tolerance and insulin sensitivity in the liver and the skeletal muscle [92]. Skeletal muscle is the primary site of insulin-mediated glucose uptake and is the main organ affected by insulin resistance and diabetes. Palmitate disrupts normal insulin signaling and alters insulin sensitivity in skeletal muscle, and MAM disorganization has been involved in the mechanism [15]. However, concomitant exposure to oleate abolishes ER stress in human myotubes [93]. Some studies have aimed at evaluating the respective roles of ER stress and oxidative stress in insulin resistance. Blockade of ER stress, but not inducible nitric oxide synthase (iNOS), restores insulin sensitivity in mouse liver and adipose tissue, whereas disruption of iNOS alone is sufficient to confer glucose tolerance in muscle [94]. The effect of ER stress on insulin signaling has been deciphered in a model of Lipopolysaccharide (LPS)-induced ER stress. IRE1/XBP1 axis induced p300, which activated acetylation of IRS1/2, thus interrupting its association with insulin receptor [95]. 

### 3.6. Mechanisms Specific to Liver Cells: Activation of Gluconeogenesis and Lipogenesis

Gluconeogenesis is an anabolic pathway of glucose synthesis from non-glucidic substrates (amino acids, lactate, and glycerol), to ensure stability of the plasma glucose level. Its role is to maintain glycemic homeostasis in conditions of low carbohydrate intake. Cyclic AMP-responsive element binding protein, hepatocyte-specific (CREBH), is a liver-specific member of the CREB/ATF family. It is an ER-resident transcription factor homolog to ATF6, and an activator of gluconeogenesis and lipogenesis in the liver. Like ATF6, its maturation occurs in Golgi after regulated proteolytic cleavage controlled by circadian oscillators [96]. CREB has been recently shown to be a key regulator of glucose level influenced by circadian rhythm and metabolic stress [97]. The emerging concept is that CREBH integrates energy metabolism with circadian rhythm. Peroxisome proliferator-activated receptor γ (PPARγ) coactivator-1α (PGC-1α) is a well-known activator of gluconeogenesis via induction of the transcription factors hepatocyte nuclear factor 4 (HNF4α) and Forkhead box protein O1 (FOXO1). XBP1s-FOXO1 interaction regulates glycemia, with FOXO1 being an activator of gluconeogenesis. XBP1s suppresses FOXO1 activity. It has been shown very recently that the physical interaction of PGC-1α with XBP1s transcription factor leads to XBP1s repression and suppresses its anti-gluconeogenic function. In this case, PGC-1α functions as a transcriptional co-suppressor [98]. Altogether, ER stress has a versatile role on gluconeogenesis, being either an activator or a repressor of this metabolic pathway, depending on which UPR branch is induced. Similar paradoxical effects on lipogenesis of XBP1s have also been observed. XBP1 was previously established as a pro-lipogenic factor [99], and, more recently, XBP1s is shown to have anti-lipogenic properties in the liver [100]. Sterol regulatory element-binding protein 1c (SREBP-1c) is a central regulator of lipogenesis. Its inactive form is located in the ER membrane, and upon activation, it is cleaved and translocated into the nucleus to act as a lipogenic transcription factor. Recent data show that the phospholipid composition of ER affects SREBP-1c maturation in both physiological and disease states [101]. This provides an insight into the regulation of glucose and lipids metabolism by ER in liver in response to lipotoxic stress. In conclusion, ER emerges as a central energy sensor and metabolic regulator in liver, and these processes seem to be finely tuned in response to specific signals as divergent effects have been unveiled.

### 3.7. Mechanisms Specific to Beta-Cells: Accumulation of Proinsulin and Amyloid Deposits in the ER

In beta cells, besides glucotoxicity, lipotoxicity, and inflammation, two other mechanisms have been described to explain the presence of ER stress in insulin resistance and diabetes: the accumulation of proinsulin and amyloid deposits in the ER. Similarly to plasma cells secreting high levels of immunoglobulins, beta-cells have a well-developed ER to ensure a high secretion of insulin. Insulin is secreted in response to glucose, a phenomenon called glucose-stimulated insulin secretion (GSIS). Under overnutrition, excessive stimulation of beta-cells challenges the folding capacity of ER and leads to accumulation of proinsulin in ER, as well as blockage of export towards the Golgi apparatus. This leads to UPR, triggering consequent apoptosis via CHOP transcription factor mediated through PERK/eIF2α/ATF4 [102]. A relevant murine model has been developed long ago to replicate proinsulin accumulation in the ER. The Akita diabetic mouse model carries a mutation in the insulin 2 gene, which substitutes a tyrosine to a cysteine normally involved in one of the three disulfide bonds linking the two chains of mature insulin. This spontaneous substitution triggers improper proinsulin folding in the ER of beta cells ER, and leads to accumulation of the mutant insulin and ER stress, thus impairing cell function and survival [103]. One of the best known discoveries made using this model is the identification of ER chaperone GRP170 involved in proinsulin degradation via ERAD in the mutant insulin *INS* gene-induced diabetes of youth (MIDY) in humans [104].

It was reported, more than a century ago, that hyaline deposits in β-cells may play a role in type 2 diabetes [105]. These amyloid deposits contain the 37-amino-acid polypeptide amylin, normally co-secreted with insulin. They occur in approximately 90% of patients with type 2 diabetes and participate in beta cell failure [106]. Human islet amyloid polypeptide (hIAPP) assembles into β-sheets, oligomers, and fibrils that result in amyloidosis. The overexpression of hIAPP in transgenic mice shows that these amyloids induce the same metabolic phenotype as those in type 2 diabetes: impaired insulin secretion, insulin resistance, and hyperglycemia [107]. Some in vitro studies suggest that the toxicity of IAPP is related to the formation of a membrane channel, inducing membrane disruption and an unregulated calcium influx [108]. Therefore, the accumulation of IAPP oligomers in ER is expected to induce calcium leakage leading to ER stress. Several ER stress markers, including CHOP and XBP1s, were observed in the islets of transgenic mice overexpressing hIAPP [107]. Apoptosis was shown to occur not only via CHOP pathway, but also via IRE1 and ATF4 branches of UPR [109]. Rat pancreatic beta-cells treated with the endogenous chaperone Bip/GRP78 or with chemical chaperones (TUDCA or PBA) displayed lower ER stress and higher GSIS [110]. ER stress induces changes towards a more reducing cellular environment, which triggers amylin aggregation [111]. A very recent publication brings new insight into IAPP conformational structure requirements for the formation of pores and the cytotoxic mechanisms in diabetes [112]. IAPP-induced toxicity eventually triggers autophagy as a response to protect against ER-related cellular damage [113,114]. The triple proline amylinomimetic compound (25, 28, 29-Pro-human amylin) named Pramlintide is a synthetic analogue of amylin currently used to treat type 1 and type 2 diabetes. It aims to increase the solubility of human amylin deposits [115]. 

### 3.8. Brain-Specific Mechanisms: Leptin Resistance

Leptin is a hormone produced by adipocytes to regulate energy homeostasis. This was the first adipokine (adipocyte-secreted cytokine) to be discovered, and it exerts its effects on hypothalamus (notably in pro-opiomelanocortin neurons) to reduce appetite. Leptin resistance, a general feature of obesity, IR, and diabetes, characterizes the inability to detect satiety despite abundant energy stores and high levels of leptin. ER stress has been proposed to be an important player in leptin resistance (recently viewed in [116,117]). Both genetic and diet-induced models of obesity are characterized by ER stress in the hypothalamus. Genetic deletion of *Ire1α* in pro-opiomelanocortin (POMC) neurons induced ER stress, as well as leptin and insulin resistance in these cells [118]. Conversely, hypothalamic GRP78 overexpression reduced ER stress and normalized metabolic parameters [119]. POMC-specific induction of XBP1s expression led to the decrease of leptin and insulin resistance, as well as diet-induced obesity even under treatment with strong activators of ER stress like tunicamycin and thapsigargin [120]. Interestingly, ER stress also plays a role in the decrease of leptin secretion by adipocytes [121]. Overall, ER stress is a general mechanism in energy homeostasis dysregulation, both at the sites of production of hormones and at the sites of reception of the hormonal signal, for leptin as well as insulin.

Altogether, it has been largely proved that ER stress plays a role in the main cell types and pathways involved in IR and diabetes; thus, it is emerging as a unifying concept explaining mechanisms underlying the genesis of diabetes and its complications.

## 4. ER and Fatty Liver Disease

Non-alcoholic fatty liver disease (NAFLD) is the most common liver disorder in developed countries. It refers to the deposition of fat within hepatocytes, due to causes other than excessive alcohol consumption. NAFLD is a general term that actually encompasses the simple deposition of fat in the liver, as well as more the progressive steatosis, non-alcoholic steatohepatitis (NASH), cirrhosis, and, eventually, cancer. NAFLD is mainly related to excess nutrients, but it is also a complex and multifactorial disease. In the United States, approximately 20% of people have NAFLD, while NASH affects between 2 and 5%. About 80% of obese individuals have NAFLD (recently reviewed in [122]). 

As for diabetes, the pathogenesis of NAFLD is closely related to obesity and insulin resistance. In fact, diabetes, fatty liver disease, and cardiovascular diseases are all related to metabolic stress induced by excessive nutrient intake and consecutive obesity. Moreover, all these diseases are related to metabolic syndrome, which is a cluster of risk factors that contribute to their development.

Relations between ER stress and insulin resistance, lipid dysregulation, inflammation, and apoptosis in the liver have already been detailed in the previous part (ER stress in IR and diabetes), but here we will focus on mechanisms linked specifically to fatty liver disease.

### 4.1. Oxidative Stress

Oxidative stress interacts with ER stress in the establishment of NAFLD/NASH (reviewed in [123]). Oxidative stress induces stress of the ER, and, conversely, ER stress stimulates the production of lipid droplets which cause the generation of ROS, thus promoting the progression of NAFLD towards NASH. Protein folding in the ER is linked to oxidative stress. Therefore, the crosstalk between ER and oxidative stress amplifies the disease progression, both being potent inducers of inflammation. Oxidative stress has been largely targeted through the use of anti-oxidant strategies in numerous attempts to reduce the production of ROS in a variety of diseases. However, several long-term antioxidant therapies have failed to attenuate the progression of the liver diseases, probably due to inefficient mitochondrial delivery and interference with normal ROS functions. One of the well-known oxidative stress response genes is nitric oxide synthase, especially its inducible isoform iNOS, which is capable of producing large amounts of NO as a defense mechanism, but it also induces inflammation. In liver, contrary to ER stress response, iNOS alone was not sufficient to account for the insulin resistance, as iNOS blockade did not improve glucose metabolism, whereas ER stress pharmacological blockade restored glucose tolerance and insulin sensitivity of the liver [94]. A key regulator of cellular resistance to oxidative stress is the transcription factor NRF2, which controls both basal and regulated expression of an array of antioxidant response element-dependent genes to regulate the physiological and pathophysiological outcomes of oxidant exposure [123]. Pharmacologic treatment with an NRF2 activator reversed some effects of NASH in the liver of WT but not *Nrf2* mutant mice. This included increased systemic insulin sensitivity, improved glucose homeostasis steatosis, and inflammation, as well as decreased steatosis and inflammation [124]. Of note, NRF2 pharmacologic activators have already been demonstrated to improve diabetes-related biomarkers [125].

### 4.2. Disturbance of Lipid Metabolism

As in the case of diabetes, numerous lines of evidence support the involvement of ER stress in the dysregulation of lipid metabolism in NAFLD. Insulin resistance leads to hepatic accumulation of triglycerides and free fatty acids. ER stress interferes with very low-density lipoprotein (VLDL) metabolism in several manners, both enhancing VLDL delivery to hepatocytes and inhibiting VLDL synthesis and export from these cells. The UPR induces VLDL receptor (VLDLR) expression via the PERK/eIF2α/ATF4 pathway, and this triggers intracellular triglyceride accumulation in the presence of VLDL [126]. A recent publication described new mechanisms of VLDLR regulation by PPARβ/δ and Fibroblast growth factor 21(FGF21). Namely, PPARβ/δ and FGF21 have a protective role against steatosis by attenuating VLDLR upregulation, and the mechanisms involved eIF2α, ATF4, and NRF2, a master regulator of cytoprotective and antioxidant response [127].

IRE1α is a key regulator in the prevention of hepatic steatosis. IRE1α was already presented as a nutrient sensor able to regulate metabolic pathways of adaptation to fasting in the liver. Mechanistically, during starvation, IRE1α induces XBP1s, which binds to and activates the promoter of PPARα, which will in turn promote mitochondrial fatty acid β-oxidation and ketogenesis as an adaption to fuel restriction [128]. Liver-specific deletion of IRE1α gene leads to modest hepatosteatosis, which becomes severe after treatment with an ER stress-inducing agent. The mechanisms include inhibition of expression of key metabolic transcriptional regulators like CCAAT/enhancer-binding protein (C/EBP) β, C/EBPδ, and PPARγ [129]. Differential effects of palmitate were shown on hepatocytes and Kupffer cells in fatty liver. Palmitate-induced ASK1 stimulation triggered cytotoxicity in hepatocytes, whereas it stimulated protective signals in Kupffer cells [130].

Besides its role in protein folding and maturation, ER also controls cholesterol synthesis and lipid-membrane biosynthesis. Excessive cholesterol can lead to ER stress and thus has an impact on the development of NAFLD/NASH (reviewed in [131]).

### 4.3. Inflammation 

Very recently, a study has shown a role of the p63/IKKβ/ER stress pathway in lipid metabolism and NAFLD. IKKβ was activated by the transactivation domain TAp63, and this induced ER stress and liver steatosis. Inhibition of ER stress attenuated the symptoms. Moreover, obese NAFLD patients displayed increased expression of TAp63, IKKβ, and XBP1s. This role of p63, a p53 ortholog in lipid metabolism and NAFLD, was not expected, although it was known to have a function in cellular adaptation to stress [132]. The IRE1-JNK pathway impaired hepatic insulin signaling transduction and triglyceride metabolism in a model of hepatic steatosis [133]. More broadly, ER stress has been assigned a role in inflammasome induction, which promotes cell death in liver. An inflammasome is a multi-molecular complex, containing Nod-like receptors (NLRs) among other proteins, and having the capacity to activate an inflammatory cascade. Different inflammasomes have been described, e.g., NOD-like receptor family and pyrin domain containing 1 (NLRP1) and NLRP3; the exact composition of an inflammasome depends on the nature of the inducer that triggered its assembly (reviewed in [134]). LPS is elevated in the plasma of patients with steatohepatitis, and it is an inducer of NLRP3 inflammasome through ER stress [135].

### 4.4. Apoptosis

In human samples of NAFLD and NASH, ER stress, apoptosis, and autophagy markers were found broadly increased, thus indicating a major dysregulation of key processes involved in protein quality control, organelle maintenance, and cell death [136]. Lipotoxicity is a potent elicitor of apoptotic cell response through PERK-dependent pathways in NASH models [137]. CHOP was necessary to mediate the beneficial effects of the glucagon-like peptide-1 (GLP-1) agonist, liraglutide, on insulin sensitivity and lipid metabolism. In *Chop*^−/−^ mice, the effects of liraglutide were extinguished [138]. Very recently, much attention has been paid to decipher the different aspects of apoptotic mechanisms in liver disease. Bax inhibitor-1 (BI-1), an inhibitor of the BCL-2 family member BAX, which is involved in apoptosis regulation, is also a negative regulator of the ER stress sensor IRE1α. *Bi-1*^−/−^ mice had increased IRE1α activity. Upon pharmacological ER stress activation, *Bi-1*^−/−^ mice showed IRE1α-dependent NLRP3 inflammasome activation and hepatocyte death. Upon high fat diet (HFD), *Bi-1*^−/−^ mice displayed activation of IRE1α, XBP1, and CHOP, as well as inflammasome and programmed lytic cell death pathway through caspase-1/-11, thus promoting NASH [139]. The ER luminal protein canopy homolog 2 (CNPY2) has been involved in PERK and CHOP activation upon ER stress-induced Bip release in the liver. Genetic deletion of the CNPY2 gene interrupted this pathway and protected hepatocytes [140].

### 4.5. Autophagy

Intersection between ER stress and autophagy has been evidenced (reviewed in [141]). Autophagy is a highly conserved self-degradative process that removes intracellular organelles via a lysosomal pathway. Autophagy has adaptive function, as it helps maintaining energy homeostasis during fasting periods [142]. Autophagy may protect cells from apoptosis in an attempt to adapt to metabolic stress by the destruction of dysfunctional components (proteins and organelles) and their recycling [143]. However, in cases of extreme and irresolvable stress, autophagy will promote apoptosis [144]. Autophagy has recently been shown to have a role in the progression of NAFLD. Autophagy is reduced both in NAFLD patients, mouse models of NAFLD, and in lipid-overloaded human hepatocytes. Thus, restoring the autophagic flux might represent a potential therapeutic way to interfere with the progression of the pathology [145]. Inhibition of CHOP decreased apoptosis for the benefit of autophagy; thus, CHOP might represent a cross point between the 2 pathways and a target for therapeutic intervention, to favor autophagy at the expense of apoptosis [146]. 

Altogether, ER stress has been assigned a role in NAFLD progression in the 2-hit model, with the 1st hit being triggered by obesity and insulin resistance and the 2nd hit by ER stress induction of inflammatory response. Less attention has been paid to ER stress involvement in NAFLD than in diabetes; thus, further investigation is needed on this topic, and this will certainly be done in the coming years.

## 5. ER Stress and Atherosclerosis

Atherosclerosis is a progressive vascular disease characterized by the deposition of lipid-laden plaques within arterial walls. It is the major cause of cardiovascular diseases, which accounts for approximately 30% of all deaths [147]. The pathology is primarily characterized by accumulation of cholesterol and oxidized low density lipoprotein (LDL) on the endothelium wall. Oxidized LDL invades the vessel wall and induces endothelial apoptosis, which subsequently leads to an inflammatory response. Monocytes adhere to the endothelium, invade it, and differentiate into macrophages, which ingest LDL and become the so-called ‘foam cells’. All these steps participate in the formation of an atherosclerotic plaque. The major complications of atherosclerosis can be attributed to thrombus formation following plaque rupture, which may occlude the artery and result in myocardial ischemia or infarction [148,149]. Despite many advances, molecular mechanisms linking cardiovascular risk factors to the development of atherosclerosis remain not fully known. 

However, the implication of ER stress in atherosclerotic plaques, particularly in the advanced stages of the disease, was found in human atherosclerotic lesions and in different animal models of atherosclerosis. Unstable atherosclerotic plaques present abnormal numbers of apoptotic cells, and this is related to ER stress [150], mainly via a robust expression of CHOP [151]. ER stress markers, such as GRP78, are strongly associated with atherosclerotic plaques in human coronary artery lesions [152]. 

Moreover, the protective effect of the chemical chaperone 4-PBA against atherosclerosis in apolipoprotein E deficient (*ApoE*^−/−^) mice proved the implication of ER stress in atherosclerosis development [153]. 4-PBAtreatment attenuated the progression of atherosclerosis and stabilized existing atherosclerotic lesions in low-density lipoprotein receptor deficient (*Ldlr*^−/−^) mice exposed to a high-fat diet [154]. One of the suggested mechanisms linking ER stress to the development of atherosclerosis is mediated by the activation of glycogen synthase kinase (GSK)-3α/β [155]. 

The major cell types of atherosclerosis include macrophages, vascular smooth muscle cells (VSMCs), and endothelial cells (ECs). Significant progress has been made in order to elucidate the roles of ER stress in these three cell types.

### 5.1. ER Stress in Macrophages

Under normal conditions, low density lipoprotein (LDL) particles are loaded from late endosomes to the ER, where cholesterol is esterified and forms inert lipid droplets. In atherosclerotic macrophages, the re-esterification of cholesterol in the ER is markedly reduced, leading to heavy deposits of non-esterified cholesterol in macrophages [156,157]. Consequently, ER oxidoreductases oxidize cholesterol to 7-ketocholesterol, which accumulates with free cholesterol and activates ER stress-induced macrophage apoptosis [158]. ER stress was previously proposed to promote proatherogenic macrophage lipid accumulation via a PERK/GSK-3α/β/CHOP pathway [159]. *Ldlr*^−/−^ mice fed a HFD represent a model for the development of atherosclerosis plaques. Pharmacological attenuation of ER stress by 4-PBA in this model reduced the signs of atherogenesis. The same results were obtained with valproate, a GSK-3α/β inhibitor, suggesting that ER stress simulates progression of atherosclerosis through GSK-3 [154].

Free cholesterol-induced apoptosis in macrophages is decreased, with IRE1 silencing [160] proving the implication of ER stress. Moreover, in human lesions and atherosclerotic plaques of *ApoE*^−/−^ mice, apoptosis is robustly associated with CHOP expression [152,161,162,163]. In fact, CHOP inactivation in *ApoE*^−/−^ mice and genetic silencing in vivo and in vitro decreased plaque rupture and macrophage apoptosis [162,164]. The mechanism linking CHOP to macrophage death involves the activation of Fas, the release of apoptogens from mitochondria, and the depletion of ER-associated calcium stores [165]. CHOP expression is also associated with a decreased expression of the anti-apoptotic protein BCL-2, leading to macrophage apoptosis in vivo [166]. 

Sometimes, ER stress is not the only cell death-inducer in macrophages. The activation of pattern recognition receptors (PRRs) may also be required to initiate apoptosis such as scavenger and toll-like receptors [167]. In plaque macrophages, PRR activation by oxidized lipids may lead to the activation of the cluster of differentiation 36-Toll-like Receptor 2 (CD36-TLR2) pathway and consequently to apoptosis, accompanied by NADPH oxidase upregulation [168]. Upregulated NADPH oxidase increases ER stress-mediated death by stimulating PERK-CHOP-dependent mechanism aggravating the apoptotic process. Recently, Ying et al. found that in the setting of a high-fat diet, perivascular adipose tissue (PVAT) accelerated plaque progression and increased atherosclerosis risk via ER stress. ER stress in PVAT destabilizes atherosclerotic plaques by activating NFκB, leading to an increased expression of granulocyte macrophage colony stimulating factor (GM-CSF) [169]. There is an increasing demand for the natural therapeutic strategies to modulate ER-induced apoptosis in atherosclerosis. Recently, a Korean group studied the effect of Kimchi, a Korean fermented vegetable dish made with cabbage, red pepper, garlic, ginger, green onion, and fermented fish sauce, on atherosclerosis occurrence in macrophages. Interestingly, they found that the antioxidant activity of quercetin in kimchi methanol extracts might retard the development of atherosclerosis through inhibition of ER stress and apoptosis [170].

### 5.2. ER Stress in Endothelial Cells (ECs)

Since the endothelium forms the final barrier of separation between the atherosclerotic plaque and the vessel lumen, ECs play a critical role in advanced atherosclerosis. 

Apoptosis of vascular ECs is a type of endothelial damage associated with atherosclerosis [171]. ER stress-induced apoptosis increases the risk of thrombosis and atherosclerotic complications by diminishing the barrier function of the vascular endothelium [172]. Various ER stress inducers were shown to initiate UPR through the activation of ATF6, GRP78, XBP1 [173,174,175,176], and both IRE1 and CHOP-mediated pathways in presence of oxidized phospholipids and homocysteine [177,178,179]. 

Moreover, ER stress in hyperglycemic *ApoE*-deficient mice model had a causative role in atherosclerotic plaque development and progression of diabetic atherosclerosis [180,181]. Alterations in calcium homeostasis caused by oxidative stress play a crucial role in ER stress-mediated endothelial dysfunction [182].

ER stress can induce atherosclerosis in human umbilical vein endothelial cell via Dickkopf1 (DKK1). DKK1 is a secretory glycoprotein, known to block Wnt pathway by competitively binding to receptors on the cell membrane. The presence of DKK1 resulted in enlarged and destabilized atherosclerotic lesions and increased apoptosis. The mechanism linking DKK1 to atherosclerosis involves the activation of JNK signal transduction pathway and the inhibition of canonical Wnt signaling, followed by the activation of the IRE1α and eIF2α/CHOP pathways [183]. 

Recently, a study has shown that miR-384 expression is reduced in atherosclerotic human umbilical vein endothelial cells (HUVECs). MiR-384 treatment prevented Angiotensin II-induced ER stress and apoptosis by targeting homocysteine inducible ER protein with ubiquitin like domain 1 (Herpud1) and suppressing ER stress markers including GRP78, CHOP, and IRE1 [184]. In cultured vascular smooth muscle cells, ER stress induced yes-associated protein 1 (YAP1), a transcriptional regulator of cell proliferation and death, which stimulated apoptosis through CHOP and caspase-3 activation [185]. Primary HUVECs treated by oxidized LDL, known to play an important role in atherosclerosis, underwent apoptosis through PERK/eIF2α/CHOP pathway [186].

### 5.3. ER Stress in Vascular Smooth Muscle Cells

The mechanisms of ER stress-mediated apoptosis in VSMCs are significantly less studied compared to those of macrophages and endothelial cells. Apoptosis of VSMCs may lead to plaque instability because of a decreased collagen production and consequent thinning of the protective fibrous cap [187]. Several ER stress-inducers have been identified in VSMCs; for example, increasing CHOP expression was associated with mechanical stretch, and treatment with 7-ketocholesterol, unesterified cholesterol, or homocysteine [188,189,190,191].

Atherosclerotic risk is known to increase in humans and animal models in the presence of elevated plasma homocysteine [191,192]. Hyperhomocysteinemia is believed to induce ER stress through upregulation of sterol response element binding protein-2 (SREBP-2), leading to an increase in lipid deposits in VSMCs and alterations of calcium balance [193,194,195]. A recent study revealed that ALDH2, a key mitochondrial enzyme in the metabolism of aldehydes, may slow the progression of atherosclerosis via the attenuation of ER stress and apoptosis in VSMCs. An in vitro model of atherosclerosis consisting of rat VSMCs treated with oxygenized low-density lipoprotein (ox-LDL), presented elevated levels of ER markers such as GRP78, PERK, p-eIF2α, ATF4, and CHOP. All the ox-LDL-induced responses were attenuated in the presence of Alda-1 (an ALDH2 activating agent) and accentuated in the presence of daidzin (an ALDH2 inhibitor) [196].

Altogether, despite the beneficial functions of the UPR during transient ER stress, pathologically chronic ER stress induces inflammation and eventually apoptosis, thus contributing to the initiation and progression of atherosclerosis. Significant progress has been made in the recent years in the management of atherosclerosis; however, new approaches are needed in this field, and ER stress may represent a novel target for therapeutic intervention. Excessive ER stress can promote the progress of atherosclerosis, mainly by inducing apoptosis in macrophages, vascular smooth muscle cells, and endothelial cells. The relationships between the UPR and the three cell types’ apoptosis in vivo are still being investigated in the hope of finding promising therapeutic targets.

## 6. Therapeutic Intervention on ER Stress in Metabolic Diseases

**Figure 5:**
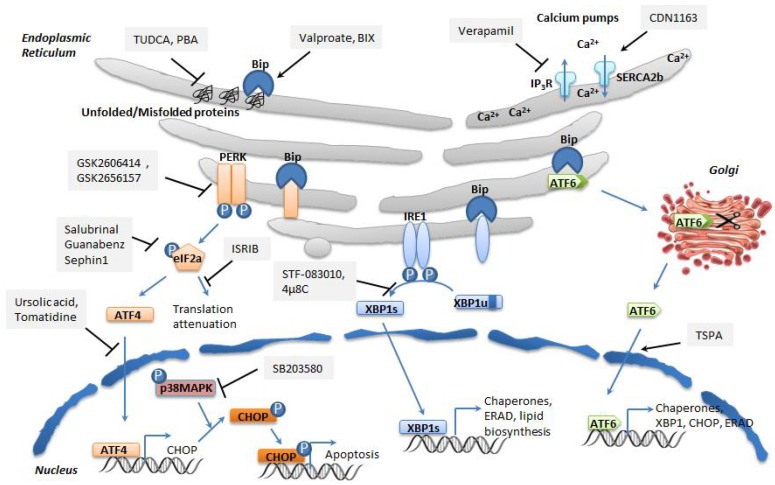
Targeting ER stress and the UPR with chemical and pharmacological drugs in metabolic diseases. TUDCA and PBA are chemical chaperones that improve protein folding in a non-specific manner. Valproate and BIX Bix are two small molecules, BIP Bip inducers. Verapamil is a calcium channel blocker, which includes the calcium release channel IP3R. CDN1163 is a small allosteric activator of SERCA2b. GSK2606414 and GSK2656157 are PERK inhibtors. Salubrinal is a small molecule, which reduces dephosphorylation of eIF2α. Ursolic acid and tomatidine are inhibitors of ATF4. SB203580 is a specific inhibitor of the p38 MAP kinase, which phosphorylates CHOP. STF-083010 and 4µ8C are inhibitors of IRE1 RNase activity. The small molecule 2-[5-[1-(4-chlorophenoxy)ethyl]-4-phenyl-4*H*-1,2,4-triazol-3-yl]sulfanyl-*N*-(1,5-dimethyl-3-oxo-2-phenyl-2,3-dihydro-1*H*-pyrazol-4-yl)acetamide (TSPA) is a small allosteric activator, which functions as an ATF6α translocation inducer. Abbreviations: inositol 1,4,5-trisphosphate(IP3R) receptorIP3R (inositol 1,4,5-trisphosphate) receptor. Grey boxes contain the name of the drugs (sharp arrows, activators; bar-ended arrows, inhibitors).

ER stress targeting has been proven to be efficient in amelioration of cell dysfunction in many metabolic diseases, as molecular chaperone approaches are successful in both experimental models, as well as in certain humans. Thus, many chemical or pharmacological drugs have been developed to target individual steps of ER stress. In general, chemical drugs exert non-specific effects on ER proteins, whereas pharmacological molecules bind to specific targets. Here, we will describe the different classes of drugs that were developed against ER stress. Dozens of molecules have already been tested, and we will only present a selection of some of the most widely used drugs as promising candidates for therapeutic purpose (Figure 5).

### 6.1. Regulators of ER Calcium Homeostasis

Calcium is a secondary messenger in numerous signal transduction cascades and regulates many enzymes and proteins. This ion has pleiotropic roles in contraction, metabolism, and trafficking, but also proliferation, differentiation, gene transcription, and apoptosis. Cytoplasmic Calcium concentration is normally low (around 100 nM), and this is achieved through active Calcium pumping from cytosol to ER, a Calcium storage compartment with up to 1000 fold higher Calcium level. Calcium homeostasis is vital to cells and especially to excitable and secretory cell types like insulin-secreting beta cells. This equilibrium relies on a high number of calcium-ATPases pumps: three SERCAs and four plasma membrane (PMCA) pumps have been discovered long ago, and two Secretory Pathway Calcium ATPase (SPCA) pumps have been unraveled more recently (reviewed in [197]). The proper function of all these pumps is crucial to calcium homeostasis, which is necessary for normal ER function, notably protein folding, modification, maturation, and trafficking. Disturbance of calcium homeostasis has been linked to ER stress-related metabolic pathologies [198]. Two complementary therapeutic strategies have been proposed to reduce calcium leakage from the ER and to ameliorate ER calcium homeostasis: blocking calcium efflux or stimulating calcium influx. 

Calcium channel blockers have been largely used to inhibit ER Calcium efflux. Blocking Calcium channel by antihypertensive drugs or muscle relaxant inhibits ER calcium efflux and induces a subset of molecular chaperones, enhancing thus overall ER protein folding capacity [199] A study using human islet cells and murine models for type 2 diabetes demonstrated that verapamil, a calcium channel blocker, inhibits the expression of the proapoptotic genes, thereby enhancing β-cell survival and preventing diabetes [200]. 

Stimulation of calcium influx has been tested through activation of SERCA pumps. The three human SERCA genes encode up to 10 isoforms by alternative splicing, with different tissue-specific expression patterns, ligands, regulations, and activities. The importance of SERCA pumps in ER stress is illustrated by the fact that thapsigargin, the widely used pharmacologic ER stressor, inhibits SERCA2b activity. SERCA2b is the major isoform expressed in smooth muscle and non-muscle tissues. SERCA2b is dramatically decreased in the liver of obese mice compared to controls. Adenovirus-mediated hepatic overexpression of SERCA2b alleviates ER stress and ameliorates glucose and lipid homeostasis [201]. Enhanced SERCA2b activity decreased both ER stress and NAFLD symptoms [202]. Small allosteric activators of SERCA2b ameliorate the symptoms of type 2 diabetes in mouse [203]. In a hepatic steatosis cell model, siRNA-mediated protein kinase C (PKC)δ inhibition stimulated SERCA activity, thereby decreasing ER stress and, notably, Bip and CHOP expression [204].

### 6.2. Non-Specific Protein Misfolding Inhibitors

Different chemical compounds such as ligands, protein inhibitors, and cofactors exert chemical chaperoning effects and are thus able to rescue folding defects, minimizing or partly averting the pathological consequences of protein misfolding [205]. These protein folding defects can be corrected by non-specific chemical chaperones such as sugars or amino acids with undefined mode of action [205]. In many circumstances, decreasing the populations of protein misfolding below certain thresholds may be sufficient to prevent either loss of function or gain of toxic function of the relevant proteins [206]. Two chemical chaperones have become the “standard of treatment” in experimental manipulation of ER stress: TUDCA, a hydrophilic bile salt which acts through binding to solvent-exposed hydrophobic segments of proteins, and 4-PBA, a terminal aromatic substituted fatty acid. They both exert a nonselective negative effect on protein aggregation and misfolding. These chemical chaperones have been proven safe for use in human; they are approved by Food and Drug Administration (FDA) for the treatment of biliary cirrhosis and urea cycle disorders, respectively. Trimethylamine N-oxide (TMAO) is another chemical chaperone developed recently, but unlike PBA and TUDCA, it is not considered as a gold standard for the reversal of ER stress [207].

As mentioned before, PBA and TUDCA are able to normalize hyperglycemia in a murine model of diabetes and restore β cell functionality in humans. Sodium phenylbutyrate, a drug with known capacity to reduce ER stress, partially alleviates lipid-induced insulin resistance and beta-cell dysfunction in humans [208]. They also mitigate hepatosteatosis in mouse models of obesity and diabetes. Atherosclerosis was reduced in *ApoE*-deficient mice treated with PBA, and macrophage apoptosis was decreased [153]. The anti-aggregation effect of TUDCA has also been used in other aggregation diseases like Alzheimer’s disease [209].

### 6.3. Bip Activators

The ER-resident protein chaperone Bip has also received attention for the development of innovative drugs, as assessed by reports on two small molecule Bip inducers, valproate, and Bip inducer X (Bix). However, despite being developed more than ten years ago, Bix has only been used in neuronal disorders and more rarely in osteoporosis. Valproate is approved for the clinical treatment of several neuronal disorders, and has been used in beta cells to protect them from palmitate-induced ER stress apoptosis [210]. However, it is also an inhibitor of histone deacetylases (HDAC) and GABA transaminase, and displays teratogenic effects.

### 6.4. Regulators of the PERK/eIF2α/ATF4/CHOP Branch 

Small molecule inhibitors of PERK and eIF2α have been considered as attractive tools for therapeutic intervention targeting ER stress in several diseases. GSK2606414 was the first PERK inhibitor discovered. It was tested in cancer and neurodegenerative disorders in mice. From this initial lead, compound was then derived a molecule with optimized pharmacokinetics named GSK2656157, which became a preclinical development candidate [211]. The two compounds have been commonly used to block ER stress; however, more recently they have been shown to repress Receptor-interacting serine/threonine-protein kinase 1 (RIPK1), a kinase mediating apoptosis, thereby pointing out a risk of misinterpretation when using these agents and a potential risk of non-specific effects in clinical use [212]. A chemical compound named integrated stress response inhibitor (ISRIB) was found to inhibit the UPR [213] and to be an activator of eIF2B. It was used to target the PERK branch of the UPR in order to inhibit ER stress-induced inflammation [214]. It was mainly used in neurological studies, and its therapeutic potential in metabolic diseases has to be evaluated.

Specific inhibitors of phosphorylation or dephosphorylation of eIF2α have also been developed. Among those, salubrinal is a small molecule that reduces dephosphorylation of eIF2α and protects cells from ER stress-induced apoptosis. It is widely used experimentally, but unfortunately has not demonstrated safety for clinical use. Several compounds were developed more recently than salubrinal. Guanabenz is a small molecule that targets eIF2α phosphorylation and thus reduces protein production and thereby ER stress [215]. Sephin1 (selective inhibitor of a holophosphatase) is a selective inhibitor of protein phosphatase 1, which has a good safety profile. It was tested with success in neurodegenerative diseases involving misfolded proteins [216]. Thus, it represents a promising candidate for ER stress management in metabolic diseases.

Attempts to block ATF4 nuclear translocation or transcriptional activity have also been reported. Two ER stress-reducing compounds, ursolic acid and tomatidine, turned out to be inhibitors of ATF4 activity in muscle diseases, but it is not clear whether it is a direct or indirect effect [217].

Targeted gene disruption of CHOP in the Akita mouse model counteracts the progression of diabetes, leading to the idea that CHOP inhibition may be beneficial to diabetes [218]. CHOP is activated through p38 Mitogen-activated protein kinase (MAPK)-mediated phosphorylation. Thus, a specific inhibitor of p38 MAPK has been used to indirectly inactivate CHOP [219]. High throughput screening has been used to discover pharmacologic inhibitors of CHOP (bioassay record AID 2732). Specific inhibitors of CHOP may be valuable in therapeutic intervention; however, more effort is needed to achieve this goal.

### 6.5. Regulators of the IRE1α/XBP1 Branch

Upon UPR activation, two IRE1α molecules oligomerize, allowing for their transautophosphorylation and endoribonuclease activation, followed by splicing of XBP1. Blocking IRE1α or XBP1 activities has proved beneficial in several diseases. Two drugs with similar structures, STF-083010 and 4µ8C, have been developed. They selectively inhibit IRE1’s RNase function by interacting with a specific lysine positioned in the active site of the IRE1 RNase domain. In an *ApoE*-deficient mouse model of atherosclerosis, these two drugs counteracted ER stress-induced inflammation in macrophages. Treatment of human peripheral blood monocytes (PBMCs) with 4µ8C led to the same findings [220]. Both drugs have demonstrated drug safety profile in humans, and are thus promising candidates for clinical use. Moreover, another strategy using IRE1α kinase inhibitors demonstrated protection of cells from ER stress and apoptosis [221,222]

### 6.6. Regulators of the ATF6 Branch

ATF6 selective modulators are the most recent drugs developed against ER stress. A small molecular compound, which is a structural analogue of other molecules with antipyretic and analgesic effects, induces ATF6α nuclear translocation without the activation of CHOP and restores insulin sensitivity in type 2 diabetes [223]. This molecule is identified as a novel lead compound and deserves further studies to evaluate its potential in other metabolic diseases.

In conclusion, the emergence of ER stress as a central mechanism in metabolic diseases and the recent drug discovery efforts in this field have led to the development of an array of small molecules to target ER stress, and their usefulness has already been demonstrated by preclinical and clinical studies. Non-specific drugs that increase general protein folding have proved to be useful, and new therapies specific to each branch of the ER stress are currently being developed. This is necessary, because each branch carries out specialized functions in a given metabolic disease, as it has been emphasized in this review. The research effort is incredibly dynamic in this relatively new field and will undoubtedly release again novel and promising candidates. These molecules will have to be tested in each distinct metabolic disease.

Besides the molecules mentioned above, regulators of oxidative stress, inducers of autophagy, inhibitors of ER stress-induced apoptosis, and protectors of mitochondria have also been proposed. More recently, therapeutic strategies have targeted SIRT1 and PPAR, the key regulators of cellular metabolism in reaction to stressors and in close relation with ER stress, in the context of metabolic diseases [224,225]. SIRT1 deacetylated eIF2α on lysine K143 and thereby protected cells from ER stress-induced injury in a model of cardiovascular disease [226]. In a HFD mouse model, PPAR agonists alleviated ER stress and improved insulin sensitivity [227]. More broadly, some of the anti-aggregation molecules developed for ER stress might be useful for the treatment of other aggregation-related pathologies like Alzheimer’s disease, Parkinson’s disease, or prion diseases. In the same way, specific ER-targeting drugs might help in the treatment of non-metabolic ER-related pathologies, e.g., inflammatory bowel disease, cystic fibrosis, and other protein misfolding disorders. It is, however, important to notice that ER stress manipulation may have undesired effects when used systemically and in the long term.

## 7. General Conclusions

With abundant nutrient supply that characterizes the modern lifestyle, metabolic diseases linked to imbalanced diet have become a common threat to human health. Metabolic derangement in part is sensed by ER, which sets up the UPR to combat the metabolic stress. Initially viewed mainly as a component of the secretory pathway, a site of lipid synthesis, and a calcium tank, ER now emerges as a key sensor and signal integrator of cellular stress capable of regulating vital metabolic pathways and the inflammatory response, and deciding the final outcome, either survival or death, of the cells. Consistent with its diverse functional roles, ER is not a homogenous entity but has many specialized domains like MAMs, ER-Golgi, and ER-plasma membrane contacts in direct communication with many other organelles. ER UPR thus recruits many non-ER organelles such as autophagosome and mitochondria to fight against cellular stress. Depending on the severity and the duration of the stress, the UPR can activate adaptive (chaperones and ERAD genes), alarm (NFκB and other inflammatory response genes), and/or cell death (caspases) responses. For these purposes, the UPR controls 3 branches of signaling pathway to fine tune the cellular outcomes. Thus, it is important to understand all this complexity to design new therapies targeted towards each metabolic disease. 

The evidence that ER stress is involved in the pathogenesis of metabolic pathologies has grown during the past few years, and the number of diseases involving ER stress has also increased, with many of them bearing similar molecular manifestations of oxidative stress, inflammation, lipotoxicity, and glucotoxicity. The UPR rescues the cells from damage caused by ER stress, and in case of persistent stress, the UPR induces apoptosis to eliminate cells with unresolvable stress. Certain ER targeted therapies aim to stimulate ER-induced autophagy rather than apoptosis to eliminate damaged proteins and organelles and rescue cell homeostasis. Therapeutic tools targeting ER stress and UPR have already proven efficient in several pathologies, leading to the proof-of-concept that ER stress can be used to manage metabolic diseases. Some of these compounds, for example, the chemical chaperones TUDCA and PBA, have been approved for clinical use despite being rather non-specific. More effort is thus needed to discover pharmacological molecules targeting specifically the ER pathways, as current therapeutic agents remain to be improved. This is the case for the currently available agents targeting CHOP or SERCA; therapeutic agents targeting other members of the UPR such as PERK inhibitors also suffer from the similar problem of non-specificity, inhibiting additional kinases like the apoptosis-mediating kinase RIPK1. However, independent of these difficulties, we believe that a universal therapeutic strategy targeting ER stress is possible, as ER stress is a shared pathomechanism of these multi-organ metabolic diseases. This is especially hopeful given the fact that ER stress has strong interconnections with oxidative stress and inflammation in the metabolic diseases described here.

## Abbreviations

4-PBA	4-phenylbutyrate
ALDH	Aldehyde dehydrogenase
AMPK	AMP-activated protein kinase
AP1	Activator protein 1
ApoE	Apolipoprotein E
ASK1	Apoptosis signal-regulating kinase
ATF	Activating transcription factor
ATP	Adenosine triphosphate
BCL	B-cell lymphoma
BI-1	Bax inhibitor-1 (BI-1)
C/EBP	CCAAT/enhancer-binding protein
CD36	cluster of differentiation 36
CNPY2	Canopy homolog 2
CREBH	Cyclic AMP-responsive element binding protein, hepatocyte-specific
Cyt c	Cytochrome c
DKK1	Dickkopf1
ECs	Endothelial cells
eIF2α	Eukaryotic initiation factor 2α
ER	Endoplasmic Reticulum
ERAD	ER-associated degradation
FA	Fatty acids
FDA	Food and Drug Administration
FGF21	Fibroblast growth factor 21
FOXO1	Forkhead box protein O1
GABA	Gamma-Aminobutyric acid
GADD153 or CHOP	C/EBPα-homologous protein
GLUT	glucose transporter
GM-CSF	Granulocyte macrophage colony stimulating factor
GRP78	78 KDa glucose-regulated proteins
GSIS	Glucose-stimulated insulin secretion
GSK	Glycogen synthase kinase
HDAC	Histone deacetylases
Herpud1	Homocysteine inducible ER protein with ubiquitin like domain 1
HFD	High fat diet
hIAPP	Human islet amyloid polypeptide
HNAs	Hydroxynaphthoic acids
HNF4α	Hepatocyte nuclear factor 4
HSPA5	Heat shock 70 kDa protein 5
HUVECs	Human umbilical vein endothelial cells
IAPP	Islet amyloid polypeptide
IFN-γ	Interferon gamma
IGF1	Insulin-like growth factor 1
IKKβ	IκB kinase β
IL	interleukin
iNOS	Inducible nitric oxide synthase
INS1 cells	Insulin-secreting cell lines
IP3Rs	Inositol trisphosphate receptors
IR	Insulin resistance
IRE1α	Inositol- requiring enzyme 1α
IRS-1	Insulin receptor substrate-1
ISRIB	Integrated stress response inhibitor
JAK	Janus kinase
JNK	JUN N-terminal kinase
LDL	Low density lipoprotein
LPS	Lipopolysaccharide
MAM	Mitochondria-associated ER membrane
MAPK	Mitogen-activated protein kinase
MIDY	Mutant insulin INS gene-induced diabetes of youth
mTORC1	Mammalian target of rapamycin complex 1
NADPH	Nicotinamide adenine dinucleotide phosphate
NAFLD	Non-alcoholic fatty liver disease
NASH	Non-alcoholic steatohepatitis
Nfe2L1	Nuclear factor erythroid 2 like 1
NFκB	Nuclear factor κB
NLRP	NOD-like receptor family and pyrin domain
NO	Nitric oxide
NOX4	NADPH oxidase 4
Nrf1	Nuclear respiratory factor 1
PBMCs	Peripheral blood monocytes
PC	Phosphatidylcholine
PDI	Protein disulfide isomerase
PE	Phosphatidylethanolamine
PERK	RNA-dependent protein kinase (PKR)-like ER kinase
PGC-1α	Peroxisome proliferator-activated receptor gamma coactivator 1 alpha
PKA	Protein kinase A
PKC	protein kinase C
PM	Plasma membrane
PMCA	Plasma membrane Ca2+ ATPase
POMC	Pro-opiomelanocortin
PPARγ	Peroxisome proliferator-activated receptor γ
PVAT	Perivascular adipose tissue
RIPK1	Receptor-interacting serine/threonine-protein kinase 1
RORα	receptor-related orphan receptor α
ROS	Reactive oxygen species
SERCA	Sarco-endoplasmic reticulum Ca(2+)-ATPase
SIRT3	Sirtuin-3
SPCA	Secretory Pathway Calcium ATPase
SREBP	Sterol regulatory element-binding protein
STAT	Signal transducer and activator of transcription
T1D	Type 1 diabetes
T2DM	Type 2 diabetes mellitus
TA	Transactivation domain
TCA cycle	Tricarboxylic acid cycle
TLR2	Toll-like Receptor 2
TMAO	Trimethylamine N-oxide
TNFα	Tumor necrosis factor alpha
TRAF2	TNF receptor–associated factor 2
TUDCA	Tauroursodeoxycholic acid
uORF	Upstream Open Reading Frame
UPR	Unfolded protein response
VLDL	Very low-density lipoprotein
VLDLR	VLDL receptor
VSMCs	Vascular smooth muscle cells
XBP1	X-box binding protein 1
YAP1	Yes-associated protein 1
